# *A C*omputerized *Co*gnitive behavioral therapy *R*andomized, Controlle*d*, pilot trial for insomnia in *P*arkinson *D*isease (*ACCORD-PD*)

**DOI:** 10.1186/s40734-017-0062-2

**Published:** 2017-08-21

**Authors:** Shnehal Patel, Oluwadamilola Ojo, Gencer Genc, Srivadee Oravivattanakul, Yang Huo, Tanaporn Rasameesoraj, Lu Wang, James Bena, Michelle Drerup, Nancy Foldvary-Schaefer, Anwar Ahmed, Hubert H. Fernandez

**Affiliations:** 1 9500 Euclid Ave/U2, Cleveland, OH 44195 USA; 2 9500 Euclid Ave/JJN3, Cleveland, OH 44195 USA; 3 9500 Euclid Ave/P57, Cleveland, OH 44195 USA; 4 9500 Euclid Ave, Cleveland, OH 44195 USA

## Abstract

**Background:**

Parkinson disease (PD) is associated with a high prevalence of insomnia, affecting up to 88% of patients. Pharmacotherapy studies in the literature addressing insomnia in PD reveal disappointing and inconsistent results. Cognitive behavioral therapy (CBT) is a novel treatment option with durable effects shown in primary insomnia. However, the lack of accessibility and expense can be limiting. For these reasons, computerized CBT for insomnia (CCBT-I) has been developed. The CCBT-I program is a 6-week web-based course consisting of daily “lessons” providing learnable skills and appropriate recommendations to help patients improve their sleep habits and patterns.

**Methods:**

We conducted a single-center, pilot, randomized controlled trial comparing CCBT-I versus standardized sleep hygiene instructions to treat insomnia in PD. Twenty-eight subjects with PD experiencing insomnia, with a score > 11 on the Insomnia Severity Index (ISI) were recruited. Based on a 6-point improvement in ISI in treatment group when compared to controls and an alpha = 0.05 and beta of 0.1 (power = 90%) a sample size of 11 patients (on active treatment) were required to detect this treatment effect using a dependent sample t-test.

**Results:**

In total, 8/14 (57%) subjects randomized to CCBT-I versus 13/14 (93%) subjects randomized to standard education completed the study. Among completers, the improvement in ISI scores was greater with CCBT-I as compared to standard education (−7.9 vs −3.5; *p* = 0.03). However, in an intention-to-treat analysis, where all enrolled subjects were included, the change in ISI between groups was not significant (−.4.5 vs −3.3; *p* = 0.48), likely due to the high dropout rate in the CCBT-I group (43%).

**Conclusion:**

This pilot study suggests that CCBT-I can be an effective treatment option for PD patients with insomnia when the course is thoroughly completed. High drop-out rate in our study shows that although effective, it may not be a generalizable option; however, larger studies are needed for further evaluation.

## Background

Parkinson Disease (PD) is associated with a high prevalence of sleep complaints, including insomnia, daytime sleepiness, sleep apnea, restless legs syndrome (RLS) and REM sleep behavior disorder (RBD) [1, 2]. The most common sleep disturbance in patients with PD is sleep maintenance insomnia, affecting up to 88% of patients [3, 4]. Sleep maintenance insomnia is characterized by a decrease in total sleep time (TST) and an increase in the number of arousals and awakenings after sleep onset. Sleep initiation insomnia affects 23% to 30% of PD patients [5, 6]. However, in controlled studies, the prevalence of sleep initiation insomnia was found to be comparable among PD patients and healthy elderly controls.

Insomnia therapy in PD has primarily consisted of pharmacotherapy using non-benzodiazepine hypnotics, sedating antipsychotics (such as quetiapine), and benzodiazepines. However, side effects, tolerance and dependency, cognitive impairment, and decreased effectiveness over time limit their use. Cognitive behavioral therapy for insomnia (CBT-I) has been shown to be more effective than pharmacotherapy long-term in primary insomnia cohorts and the NIH State-of-the-Science conference Statement identified it as the first-line approach to insomnia treatment [7]. CBT-I helps identify and modify negative thoughts about sleep and behaviors that perpetuate insomnia [8]. However, widespread use of CBT-I has been limited by lack of trained clinicians, geographical remoteness of the trained providers, stigmatization of receiving psychological service and expense. For these reasons, computerized cognitive behavioral therapy for insomnia (CCBT-I), has been developed in order to make CBT-I more convenient. The Cleveland Clinic CCBT-I program [9] consists of sleep restriction, stimulus control, cognitive restructuring, sleep hygiene, and relaxation training delivered in stages over a 6-week period [10–12]. The efficacy of CCBT-I for primary insomnia has been demonstrated in several randomized controlled trials [13–17].

Despite the high prevalence and life-quality impact of insomnia in PD, there are only a few pharmacotherapy studies specifically addressing insomnia without a focus on nocturnal motor symptoms [18–21]. Most recently, a three-arm six-week randomized pilot study comparing non-pharmacologic treatment (cognitive behavioral therapy/bright light therapy) versus doxepin 10 mg at bedtime versus inactive placebo, was published [22]. Although it was found that doxepin and non-pharmacologic treatment substantially improved insomnia, we are aware of no study on CCBT-I which provides near universal access to this patient population. Therefore, we have conducted a pilot study evaluating the effect of CCBT-I on insomnia in PD patients.

## Methods

This was a 6-week pilot randomized (1:1 ratio), parallel-group, controlled study evaluating effectiveness of CCBT-I in PD patients by measuring clinical and sleep variables before and after completion of the program. PD patients with insomnia were asked to participate at their clinical visit by their current movement disorder provider. If the patient was interested, the research coordinator or investigator discussed study further with patient. Subjects meeting inclusion criteria (Table 1) were randomized to CCBT-I or standard sleep hygiene education (Table 2). Randomization was done by study coordinator who made 28 sealed envelopes containing their group designation. Once patients signed the informed consent form, they were given the sealed envelope containing their group designation.

**Table 1:** Inclusion and Exclusion Criteria

*Inclusion Criteria*	*Exclusion Criteria*
1. 35–85 years of age	1. Dementia as defined by DSM-IV criteria
2. Diagnosis of PD by a Movement Disorders neurologist	2. Patients with suboptimally treated depression and significant depressive symptoms as defined by a PHQ-9 score of > 15. Antidepressant medications prescribed for depression or anxiety were allowed if the patient had been on a stable dose for at least 1 month.
3. On stable antiparkinsonian medications for the past 30 days	3. Significant hallucinations or psychotic symptoms requiring antipsychotic medications
4. ISI > 11	4. Presence of significant sleep disorders that could be contributing to insomnia such as known sleep apnea, RBD, RLS
5. Access to a computer and internet	5. Presence of significant motor fluctuations, especially nocturnal akinesias that could be contributing to insomnia
6. Be able to speak, read and understand English	6. Use of sedatives, benzodiazepines or sedating antidepressants (such as mirtazapine, TCAs), modafinil, stimulants, anticholinergic medications, were allowed if the patient had been on a stable dose for at least 1 month and was not taking it as a sleep aid.
	7. Significant renal, hepatic, cardiac and thyroid disease that could have interfered with protocol adherence

**Table 2:** Sleep Hygiene Education

BEFORE GETTING INTO BED:
-Do not eat a heavy meal close to bedtime (a light bedtime snack is OK),
-Create a positive sleep environment – cool, dark and quiet,
-Create a buffer zone – quiet time prior to bed time. During this time, you should do things that are enjoyable on their own rather than activities that are goal oriented.
WHILE IN BED:
-Avoid watching the clock. Turn your clock around (or cover it) and use your alarm if needed.
-Use your bed only for sleep and sex – Avoid TV watching, use of computer, reading, or cell phone use in bed.
IN THE MORNING AND DURING THE DAYTIME:
-Avoid naps during the day.
-Limit caffeine and consume before noon.
-Exercise regularly but not within 3–4 h of bedtime.

Subjects completed questionnaires at baseline, 8 and 12 weeks after randomization, including:

1. Epworth Sleepiness Scale (ESS) —The ESS is a 4-point scale (0–3) measuring daytime sleepiness of a patient in 8 different situations or activities that most people engage in as part of their daily lives, although not necessarily every day. The total ESS score can range between 0 and 24. The higher the score, the higher the person’s level of daytime sleepiness [23].
2. Pittsburgh Insomnia Rating Scale (PIRS20) —PIRS20 is a subjective measurement of the severity of insomnia that the patient rates [24].
3. Insomnia Severity Index (ISI) —ISI is a valid and reliable tool to diagnose and measure severity of insomnia. It consists of 7 questions concerning sleep onset, sleep maintenance, early awakening, level of satisfaction with sleep pattern, extent of interference with daily functioning, conspicuousness of impairment caused by sleep problem, and level of concern about current sleep problem. Each item is marked on a 5-point Likert scale (0 to 4). Total scores after evaluation ranges from 0 to 28; the higher score, the more insomnia severity. Scores 0 to 7 indicate no clinically significant insomnia, 8 to 14 sub-threshold insomnia, 15 to 21 clinically significant insomnia (moderate), and 22 to 28 clinically significant insomnia (severe) [25].
4. Fatigue Severity Scale (FSS) —FSS measures fatigue severity by measuring its effect on daily activities in patients with chronic neurologic disorders. It is measured on a 7-point scale (0–7), higher the score indicating more severe fatigue [26].
5. Unified Parkinson Disease Rating Scale (UPDRS) parts 1b and II—UPDRS 1b and II measure activities of daily living in PD evaluating motor and non-motor features. It is a 20-question survey, each question with a 0–4 scale, the higher the score, the more severe the impact on the particular activity. When analysing the data, we modified the score by removing the question related to sleep to evaluate if the score change in a patient’s activities of daily living that wasn’t swayed by improvement in sleep.
6. Patient Health Questionnaire (PHQ-9)—The PHQ-9 is a multipurpose instrument for screening, diagnosing, and measuring severity of depression. The score ranges from 5 to 20, with the higher score suggesting more severe depression [27].
7. Parkinson Disease Questionnaire (PDQ8)—PDQ8 is an 8-question self-administered questionnaire, used to measure quality of life in persons with Parkinson’s disease. Scores range from 0 to 4, the higher the score, the greater the impairment on the person’s quality of life. The 39-point PDQ provides scores for each of the 8 domains: mobility, activities of daily living, emotional well-being, stigma, social support, cognitions, communications and bodily discomfort. Alternatively, the sum of the domain scores can be used to assess the overall health-related quality of life profile of the individual questioned [28].

### CCBT-I therapy

Go! To Sleep is a 6-week online, interactive CBT-I based program designed to foster better sleep habits and help participants implement cognitive behavioral therapy for insomnia strategies. Subjects were provided with a unique password to access the program. Daily program access is encouraged via daily email reminders to complete a sleep log based on prior night’s sleep pattern. After completing sleep log, the user is given individualized feedback based on their sleep log responses as well as a daily sleep efficiency score to help them track their progress through the program. There are daily “lessons” or articles that provide psychoeducation regarding insomnia and strategies to help address their sleep concerns. In addition, throughout the program they have access to relaxation/meditation practices as well as other strategies designed to improve stress management and sleep. There is a mobile application for easy sleep tracking.

Table 2 shows the sleep hygiene advice that was given to the control group.

All subjects received weekly telephone reminders from an investigator to participate in their assigned therapy. Additional telephone calls were made 8 and 12 weeks after randomization to complete the questionnaires and return to the investigators in self-addressed envelopes. Subjects were considered a dropout if they could not complete CCBT-I or if they did not return the week 8 questionnaires.

The Institutional Review Board of the Cleveland Clinic approved the project.

### Statistical methods

CCBT-I has never been tested in the PD population, thus we did not have prior experience from which to perform sample size calculations. However, there is a study using ISI and advocating for a 6-point change as significant [29]. This study gives population mean value for ISI as 19.7 with standard deviation as 4.1. Another study examining the validation of the ISI as an outcome measure for insomnia research showed population mean value for ISI as 17.9 with standard deviation as 4.1 [30]. In a three-arm (cognitive behavioral therapy/bright light therapy versus doxepin versus inactive placebo), six-week, randomized study, the baseline ISI was 14.7 ± 6.1, 19.9 ± 3.7 and 16.5 ± 5.4 [22]. Based on a 6-point improvement in ISI in treatment group when compared to controls and an alpha = 0.05 and beta of 0.1 (power = 90%) a sample size of 11 patients (on active treatment) were required to detect this treatment effect using a dependent sample t-test. Assuming a dropout rate of 25%, a sample size of 14 patients on each arm were recruited.

The data are presented as mean ± standard deviation for continuous variables and N (%) for categorical variables. Comparison of demographic variables was performed by two-sample t test in continuous variables, and chi-square test or Fisher exact test in categorical variables. Change from baseline to end point was analyzed using paired t test; and compared between cases and controls by two-sample t test.

To account for the correlation among repeated measures on the same subject a mixed model assuming compound symmetry correlation structure was used to test the trend from Week 1 to Week 6 of the online program. Least square means with 95% confidence intervals were presented to show the trend. Analyses were performed based on an overall significance level of 0.05, using SAS software (version 9.4, Cary, NC).

## Results

Twenty-nine subjects were screened for enrollment. One subject was not considered a candidate after screening due to low ISI score. Subsequently, 28 subjects were randomized, 14 in each group. Sample characteristics are shown in Table 3. There were no significant differences between groups except for gender, where there were more males in the CCBT-I group than in the control group.

**Table 3:** Baseline Characteristics

	Case	Control	*p*-value
Age (years)	63.1 ± 6.8	64.7 ± 9.5	0.62^a^
Gender			***0.022*** ^***c***^
Female	3(21.4)	9(64.3)	
Male	11(78.6)	5(35.7)	
ESS Total	11.1 ± 4.9	9.3 ± 6.2	0.39^a^
FSS Total	27.3 ± 13.1	29.1 ± 12.2	0.72^a^
ISI Total	15.7 ± 3.0	15.4 ± 2.9	0.80^a^
UPDRS1b Total	9.8 ± 4.0	9.4 ± 2.1	0.71^a^
UPDRS1b Modified	5.4 ± 3.4	5.1 ± 2.0	0.79^a^
UPDRS2 total	12.6 ± 7.9	10.4 ± 7.1	0.46^a^
PDQ Total	14.4 ± 4.4	15.2 ± 5.8	0.70^a^
PHQ9 Total	7.8 ± 5.1	7.2 ± 4.2	0.73^a^
pirs20 Total	9.1 ± 2.4	9.1 ± 2.1	0.92^a^
EQ5D Index	0.75 ± 0.15	0.79 ± 0.13	0.46^a^

*p*-values: ^a^two-sample *t* test, ^c^Pearson's chi-square test

Bold values are statistically significant *p* < 0.05

Six subjects in the treatment group and 1 in the control group withdrew from the study. Using intention-to-treat analysis, the last available data point was used as endpoint.

Intention-to-treat analysis showed a significant improvement in ISI (*p* = 0.007), ESS (*p* = 0.048) and PIRS20 (*p* = 0.004) and PHQ-9 (*p* = 0.011) scores in the treatment group when compared to the respective scores prior to CCBT-I treatment, as shown in Table 4. UPDRS1b scores also significantly improved after treatment, however when modifying the score by removing the sleep variables, the change was no longer significant. However, when comparing the treatment group to the control group, none of these changes were significant, including ISI (−4.5 vs −3.3; *p* = 0.48).

**Table 4:** Baseline and endpoint scores per an Intention to Treat Analysis

	Case(*N* = 14)			Control(*N* = 14)		
Factor	Baseline	Endpoint	*P* value	Baseline	Endpoint	*P* value
ESS Total	11.1 ± 4.9	9.5 ± 5.8	***0.048***	9.3 ± 6.2	8.1 ± 4.8	0.25
FSS Total	27.3 ± 13.1	25.4 ± 13.6	0.20	29.1 ± 12.2	29.2 ± 12.6	0.70
ISI Total	15.7 ± 3.0	11.2 ± 5.6	***0.007***	15.4 ± 2.9	12.2 ± 5.1	***0.008***
UPDRS1b Total	9.8 ± 4.0	8.1 ± 4.2	***0.010***	9.4 ± 2.1	8.4 ± 2.5	***0.043***
UPDRS1b Modified	5.4 ± 3.4	5.0 ± 3.2	0.34	5.1 ± 2.0	4.9 ± 1.7	0.58
UPDRS2 total	12.6 ± 7.9	12.5 ± 7.0	0.93	10.4 ± 7.1	10.2 ± 8.0	0.77
PDQ Total	14.4 ± 4.4	14.2 ± 4.3	0.52	15.2 ± 5.8	14.6 ± 5.6	0.32
PHQ9 Total	7.8 ± 5.1	5.8 ± 5.5	***0.011***	7.2 ± 4.2	5.9 ± 4.1	0.14
pirs20 Total	9.1 ± 2.4	7.2 ± 3.5	***0.004***	9.1 ± 2.1	7.5 ± 3.9	0.070
EQ5D Index	0.75 ± 0.15	0.72 ± 0.16	0.23	0.79 ± 0.13	0.76 ± 0.16	0.49

Values presented as Mean ± SD. *p*-values: paired *t* test

Bold values are statistically significant *p* < 0.05

Per protocol analysis, i.e. only using patients who did complete treatment, is shown in Table 5. One subject in the control group who had started using a sleeping agent was excluded. A significant improvement in ISI (*p* = 0.002), ESS (*p* = 0.042), PIRS20 (*p* = 0.005) and PHQ-9 (*p* < 0.001) scores were observed in the treatment group. In addition, the change in ISI was significantly greater in the treatment group (−7.9 ± 4.5) compared to controls (−3.5 ± 3.9) (*p* = 0.033).

**Table 5:** Baseline and endpoint scores Per Protocol Analysis

	Case(*N* = 14)				Control(*N* = 14)			
Factor	n	Baseline	Endpoint	*P* value	n	Baseline	Endpoint	*P* value
ESS Total	8	11.1 ± 4.9	8.4 ± 5.9	***0.042***	12	9.8 ± 6.2	9.0 ± 4.5	0.40
FSS Total	8	27.3 ± 13.1	21.3 ± 11.0	0.21	12	30.5 ± 11.5	28.2 ± 11.2	0.35
ISI Total	8	15.7 ± 3.0	7.8 ± 4.4	***0.002***	12	15.3 ± 3.0	12.2 ± 5.5	***0.033***
UPDRS1b Total	8	9.8 ± 4.0	6.9 ± 3.1	***0.004***	12	9.7 ± 1.8	8.6 ± 2.4	***0.037***
UPDRS1b Modified	8	5.4 ± 3.4	4.4 ± 2.1	0.35	12	5.4 ± 1.7	4.8 ± 1.6	0.77
UPDRS2 total	8	12.6 ± 7.9	12.3 ± 6.3	0.93	12	10.9 ± 7.1	10.6 ± 8.4	0.99
PDQ Total	7	14.4 ± 4.4	13.7 ± 3.7	0.53	11	15.6 ± 5.8	13.1 ± 3.8	0.73
PHQ9 Total	8	7.8 ± 5.1	3.5 ± 4.5	***0.005***	12	7.5 ± 4.2	5.4 ± 3.3	0.17
pirs20 Total	8	9.1 ± 2.4	5.0 ± 2.3	***<0.001***	12	9.2 ± 2.1	7.4 ± 3.9	0.14
EQ5D Index	8	0.75 ± 0.15	0.71 ± 0.17	0.24	12	0.79 ± 0.13	0.77 ± 0.17	0.55

Values presented as Mean ± SD. *p*-values: paired *t* test

Bold values are statistically significant *p* < 0.05

Five patients had a change in their medication during the trial. Two patients in the control group reported a decrease in their PD regimen, which they felt may have helped their sleep. One patient in the control group started taking a sleeping aid. Two patients (1 in treatment and 1 in control) had an increase in antidepressant medications.

## Discussion

The etiology of insomnia in PD is heterogeneous and may arise from motor and non-motor features of PD such as nocturnal akinesia, nocturnal dystonia, wearing off, dyskinesias, nocturia, depression, anxiety, dementia, medications, punding, as well as primary sleep disorders [31, 32]. We performed the first randomized control trial to compare the effectiveness of web-based CBT-I compared to standard recommendations for insomnia in the PD population. While no significant difference in insomnia severity, as measured by the ISI, was found in the ITT analysis (likely due to the high drop out rate in the CCBT-I group), the *per protocol* analysis, amongst patients who finished the study, found significant improvement in insomnia symptoms with the CCBT-I than standard sleep hygiene education. Consistent with the improvement in ISI, subjects treated with CCBT-I also experienced significant increases in sleep efficiency that persisted to the final assessment at 12 weeks. As mentioned earlier, there are very few studies that evaluated the treatment of sleep in PD patients.

Finally, statistically significant improvements in insomnia scores observed in the control group, after receiving standard written sleep hygiene recommendations, supporting the value of simple sleep education in clinical practice. However, the degree of improvement in the ISI score was not more than 6 points to consider clinically significant improvement. This suggests that more effective treatment is still in need.

The major limitation of the study, in addition to the open-label nature of the treatment assignment, is the high dropout rate in PD population as this group had a difficult time completing the program. Dropout rates are variable across all PD clinical trials and can be up to 50%. A recently published meta-analysis on self-help CBT (via booklet, videotape, audiotape or internet) for insomnia reported an average dropout rate of 14.5% compared to 16.7% in therapist-administered CBT [33]. Our dropout rate was about 43%. While all patients had computer access, they were not used to using it on regular basis. The average age of our population was 64 years old, which is roughly 10–20 years older than prior studies that evaluated this tool for treatment for insomnia. A younger population may be more comfortable using a computer on a daily basis and therefore may achieve a higher success rate, compared to the average PD patient. Indeed, when we asked patients what were the barriers of CCBT-I that limited them from completing the program, most of them felt that the program interrupted their normal lifestyle. Patients were unable to keep up with daily logs and would have a tendency to forget to log on to computer to complete the tasks. Nonetheless, the difference in improvement of ISI scores in the *intention-to-treat* versus the *per-protocol* cohort suggests that CCBT-I treatment is only effective if patients carry it through all the way to the end.

This can be challenging for PD patients due to difficult time keeping up with daily logs and it may be helpful to decrease logging frequency to once a week. In addition, one on-line CBT-I study found that compared to physician-referred participants (46.7%), community-recruited participants were significantly less likely to drop-out (18.2%) suggesting that community based recruitment may have higher levels of pre-treatment motivation or more comfortable with technology [15]. In addition, we can consider refining the program to tailor to the unique needs PD patients, such as permitting a brief afternoon nap, if needed, to address fatigue and modification of sleep restriction and other behavioral strategies that may be more difficult for individuals with comorbid chronic health issues. The addition of periodic semi-structured video-chats or phone calls with trained personnel to monitor progress and problem-solve issues with the online program would likely decrease participant drop out as well as increase program efficacy.

In addition, 5 patients did have a change in their medication during the study. One patient, who added a sleep aid, was removed from the analysis. Changes in PD medications as well as antidepressants can certainly aid in improving sleep; however, these changes were done for PD management and treatment of depression, not necessarily to treat insomnia. Any benefit patients received in sleep is consistent with the theory that insomnia in PD population is heterogeneous and many factors are contributory.

Our observations expand the existing literature on the use of CCBT-I in clinical practice, examining its feasibility and effectiveness in an older cohort of PD patients with motor and non-motor impairments. Larger studies, with perhaps a more thoughtful construction of behavioral therapy execution and greater vigilance of its compliance, are needed to definitively compare the effectiveness on different types of insomnia therapies in PD patients.

## Conclusions

This pilot study suggest that CCBT-I can be an effective treatment option for PD patients with insomnia when the course is thoroughly completed. High drop-out rate in our study shows that although effective, it may not be a generalizable option. We can tailor this program for the unique needs of PD patients and provide more trained personnel to monitor progress and problem-solve to increase program efficacy. However, larger studies are needed for further evaluation.

## Abbreviations

CBT-I: Cognitive behavioral therapy for insomnia
CCBT-I: *computerized* cognitive behavioral therapy for insomnia
ESS: Epworth Sleepiness Scale
FSS: Fatigue Severity Scale
ISI: Insomnia Severity Index
PD: Parkinson Disease
PDQ8: Parkinson Disease Questionnaire
PHQ-9: Patient Health Questionnaire
PIRS20: Pittsburgh Insomnia Rating Scale
RBD: REM sleep behavior disorder
RLS: Restless legs syndrome
TST: total sleep time
UPDRS1b and II: Unified Parkinson Disease Rating Scale 1b and II
